# The inverse association between DNA gaps and HbA1c levels in type 2 diabetes mellitus

**DOI:** 10.1038/s41598-023-46431-2

**Published:** 2023-11-03

**Authors:** Jirapan Thongsroy, Apiwat Mutirangura

**Affiliations:** 1 School of Medicine, Walailak University, Nakhon Si Thammarat, 80160 Thailand; 2 Research Center in Tropical Pathobiology, Walailak University, Nakhon Si Thammarat, 80160 Thailand; 3 Center for Excellence in Molecular Genetics of Cancer and Human Diseases, Chulalongkorn University, Bangkok, Thailand; 4 Department of Anatomy, Faculty of Medicine, Chulalongkorn University, Bangkok, Thailand

**Keywords:** Molecular biology, Biomarkers, Molecular medicine

## Abstract

Naturally occurring DNA gaps have been observed in eukaryotic DNA, including DNA in nondividing cells. These DNA gaps are found less frequently in chronologically aging yeast, chemically induced senescence cells, naturally aged rats, d-galactose-induced aging model rats, and older people. These gaps function to protect DNA from damage, so we named them youth-associated genomic stabilization DNA gaps (youth-DNA-gaps). Type 2 diabetes mellitus (type 2 DM) is characterized by an early aging phenotype. Here, we explored the correlation between youth-DNA-gaps and the severity of type 2 DM. Here, we investigated youth-DNA-gaps in white blood cells from normal controls, pre-DM, and type 2 DM patients. We found significantly decreased youth-DNA-gap numbers in the type 2 DM patients compared to normal controls (P = 0.0377, P = 0.0018 adjusted age). In the type 2 DM group, youth-DNA-gaps correlate directly with HbA1c levels. (r = − 0.3027, P = 0.0023). Decreased youth-DNA-gap numbers were observed in patients with type 2 DM and associated with increased HbA1c levels. Therefore, the decrease in youth-DNA-gaps is associated with the molecular pathogenesis of high blood glucose levels. Furthermore, youth-DNA-gap number is another marker that could be used to determine the severity of type 2 DM.

## Introduction

Type 2 DM has been a public health issue for several decades. Currently, an estimated 537 million people are at risk of developing type 2 DM, and that number is expected to rise to 592 million by 2035 and 783 million by 2045^1,2^. A previous study demonstrated that patients with type 2 DM often have relatively impaired genetic function, and genome instability is believed to be a factor that contributes to type 2 DM^3–10^. Type 2 DM patients are more likely to develop various conditions that affect elderly people, causing type 2 DM to be one of the world's most major health problems^11–20^. Tissue damage and multiorgan dysfunction, such as cardiovascular disease, stroke, foot ulcers, chronic kidney failure, and diabetic retinopathy, can occur in type 2 DM patients^21–29^. However, the mechanism underlying cellular senescence in DM needs more research.

We previously reported the discovery of naturally occurring DNA gaps called youth-associated genome-stabilizing DNA gaps (youth-DNA-gaps)^30^. Youth-DNA-gap complexes are composed of Box A of HMGB1, which produces the DNA gap, SIRT1 deacetylated histones, and AGO4 methylated interspersed repetitive sequences^31–35^. Naturally occurring DNA gaps in the genome maintain genomic integrity, and these gaps decrease in aging yeast, rats, and human and chemically induced senescent cells^31,36–38^. The formation of DNA gaps is one of the cellular mechanisms that maintains DNA integrity. As a result, the role of youth-DNA-gaps is similar to that of gaps that remain between successive rails in a railway track, that is, relieving torsion force to prevent damage^33^. We previously demonstrated that reducing the DNA gap resulted in DNA shearing^37^. Moreover, introducing new Box A-produced DNA gaps increased DNA resistance to radiation-induced DNA breaks, rejuvenated senescent cells, and rejuvenated both naturally and d-galactose-induced aging rats^33^. Two types of DNA breaks occur in eukaryotic cells. The most common type is pathologic breaks. This type of break is associated with H2AX foci and exists is small numbers under normal circumstances^39–44^. Youth-DNA-gaps are another type of DNA break that plays a physiologic role and is produced by cellular enzymes. Therefore, while a pathologic break represents DNA damage, youth-DNA-gaps represent epigenetic markers. Although both types are detectable by PCR, we proved by high throughput sequencing that the majority of PCR-detected DNA breaks under physiologic conditions are youth-DNA-gaps^38,40^. In aging cells, pathologic breaks are actively produced; however, the overall number of DNA gaps that could be detected by DNA gap PCR is reduced^37^. Therefore, DNA gap PCR results can indicate the youthfulness of eukaryotic DNA^33,37^. Type 2 DM accelerates the aging process both at the molecular level and in terms of physical phenotypes. Patients with type 2 DM have a higher risk of developing common geriatric syndromes, including frailty, arteriosclerosis, kidney failure, and dementia ^45–52^. Similar to elderly, type 2 DM patients have increased telomere shortening, mitochondrial DNA depletion, Alu hypomethylation, and DNA methylation age epigenetic clock^53–62^. Since the number of youth-DNA-gaps is inversely associated with the degree of aging and the degree of aging phenotype is directly associated with the severity of type 2 DM, the number of youth-DNA-gaps should be low in patients with severe type 2 DM.

Presently, blood tests are used to diagnose type 2 DM by measuring fasting blood sugar (FBS) and hemoglobin A1c (HbA1c) levels ^63–69^; guidelines are used to design good diabetes care primarily based on HbA1c levels ^70,71^. However, HbA1c and FBS levels represent the excess sugar levels in blood ^71,72^; another type of molecular marker that indicates the severity of organ degeneration will help determine the prognosis of type 2 DM patients. The assessment of youth-DNA-gaps by DNA gap PCR provides information on the biological aging of the individual.

We hypothesized that a decrease in youth-DNA-gap numbers promotes the pathogenesis of type 2 DM by inducing DNA damage, leading to the cellular senescence process, and directly correlating with the disease. Therefore, reducing the youth-DNA-gap number should inversely correlate with HbA1C and might serve as a promising biomarker for monitoring and treating tissue degeneration associated with type 2 DM in the future.

## Results

### Youth-DNA-gaps in patients with type 2 DM

**Figure 1:**
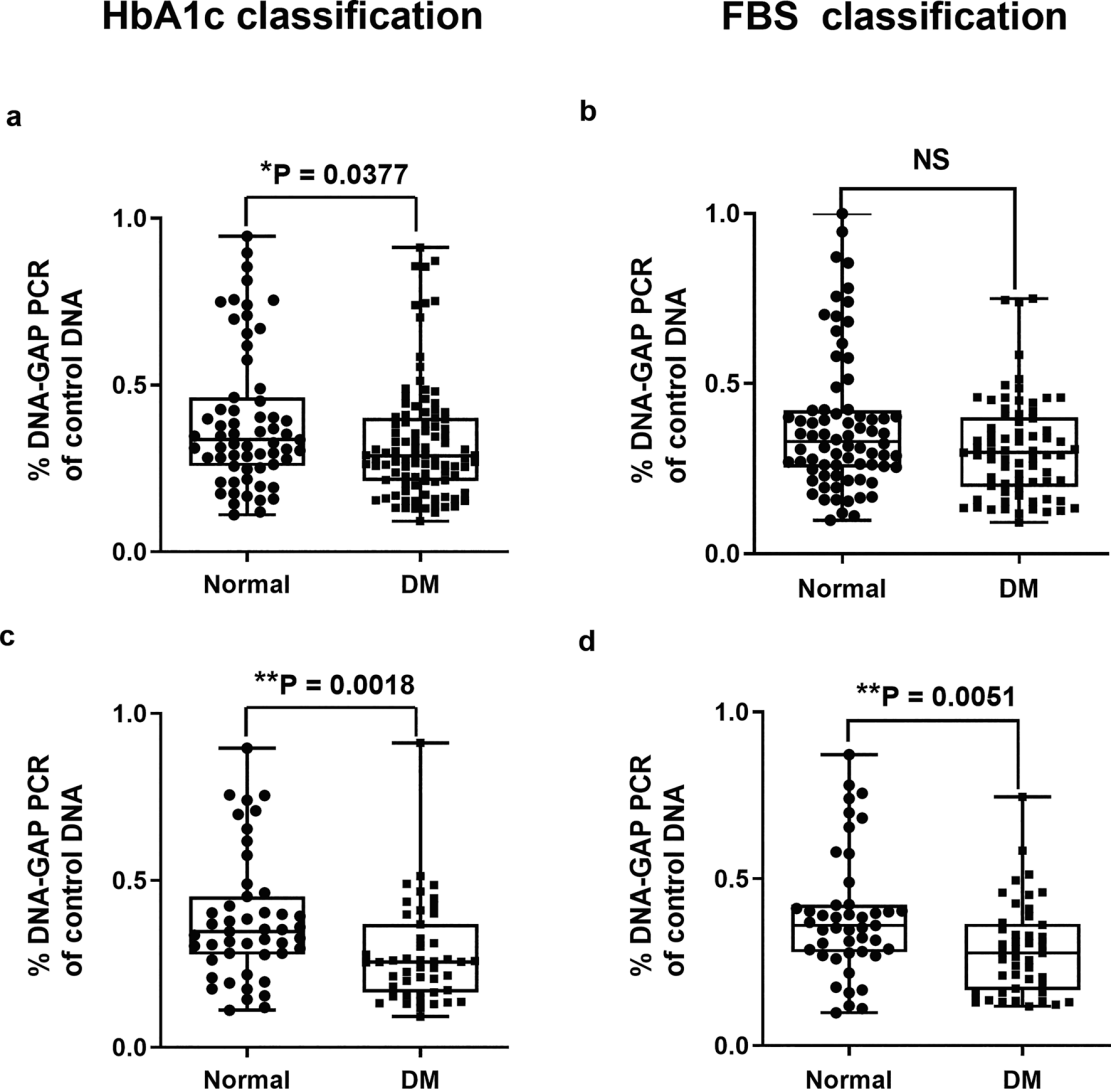
The percentage of each DNA-gap number in normal and type 2 DM patients when grouped by HbA1c (**a**) and FBS levels (**b**). The values of independent age-matched pairs when grouped by HbA1c (**c**) and FBS levels (**d**). The boxes represent the interquartile ranges (25th to 75th percentile), while the median lines represent the 50th percentile. The whiskers represent the minimum and maximum values, and all individual data points are displayed. Significance levels are denoted as follows: *P < 0.05, **P < 0.01 (Mann–Whitney U test).

The 240 samples were divided into three groups based on their HbA1c levels: 63 normal controls, 78 pre-DM, and 99 type 2 DM patients. Additionally, fasting blood sugar (FBS) levels were used to classify these patients, and as a result, several patients changed groups (74 normal controls, 93 pre-DM, and 73 type 2 DM patients) compared to the grouping based on HbA1c values (Table 1). The youth-DNA-gap numbers in each sample was then measured. We found that the number of youth-DNA-gaps was decreased in type 2 DM patients when the patients were classified by HbA1c (P = 0.0377) (Fig. 1a). We found no significant difference in youth-DNA-gap number in type 2 DM patients compared with normal subjects when the patients were grouped according to FBS levels (Fig. 1b).

**Table 1:** Sample size, gender, age, and body mass index (BMI) of each group when grouped by HbA1c and FBS levels.

	Group			P value
	Normal	Pre-DM	DM	
HbA1c indicator				
N	63	78	99	
Gender				
Male	9 (14.29%)	18 (23.08%)	22 (22.22%)	
Female	54 (85.71%)	60 (76.92%)	77(77.78%)	
Age (years) (mean ± SD)	55.30 ± 10.58	55.08 ± 9.61	58.33 ± 11.55	0.0798
BMI (kg/m^2^) (mean ± SD)	24.93 ± 3.87	26.28 ± 4.37	26.12 ± 4.82	0.0906
FBS indicator				
N	74	93	73	
Gender				
Male	13 (17.57%)	17 (18.28%)	19 (26.03%)	
Female	61 (82.43%)	76 (81.72%)	54 (73.97%)	
Age (years) (mean ± SD)	55.89 ± 10.67	55.42 ± 10.97	58.42 ± 10.48	0.0758
BMI (kg/m^2^) (mean ± SD)	25.06 ± 3.84	25.80 ± 4.17	26.73 ± 5.24	0.0615

In our data, the average age of the normal and type 2 DM groups was different (Table 1). Age-matched pairs were created by matching normal to type 2 DM patients of the same age. Age-matched pairs revealed a significantly lower youth-DNA-gap numbers in the type 2 DM group than in the normal group when the patients were grouped according to HbA1c and FBS levels (P = 0.0018 and P = 0.0051, respectively) (Fig. 1c and d, respectively).

**Figure 2:**
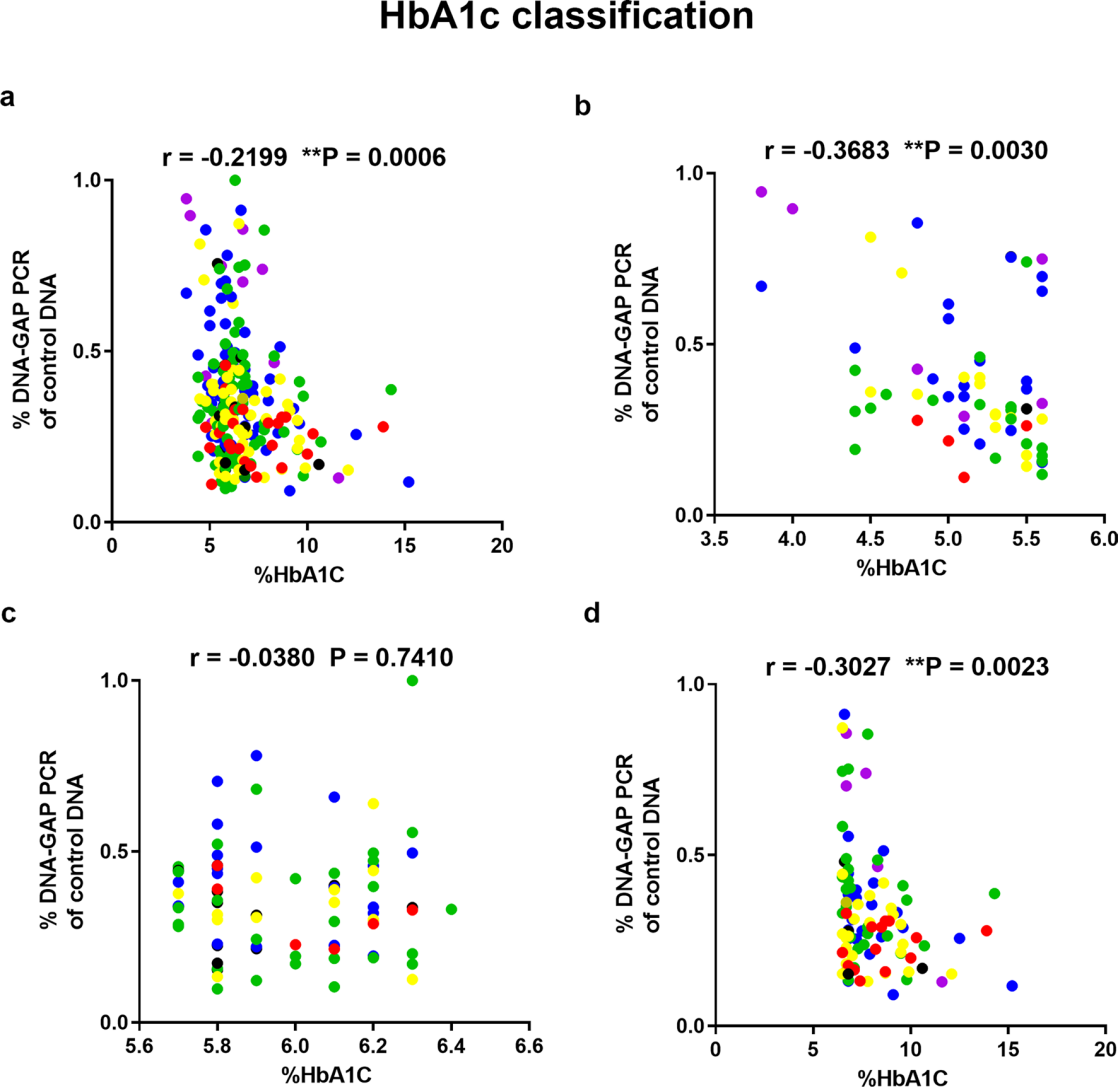
Association between the percentage of each DNA-gap with HbA1c levels in all samples (**a**), normal (**b**), pre-DM (**c**), and type 2 DM patients (**d**). Spearman's rank correlation (r) with P values are indicated. (**P < 0.01). The age ranges are represented by the following colors: violet (31–40 years), blue (41–50 years), green (51–60 years), yellow (61–70 years), red (71–80 years), and black (81–90 years).

**Figure 3:**
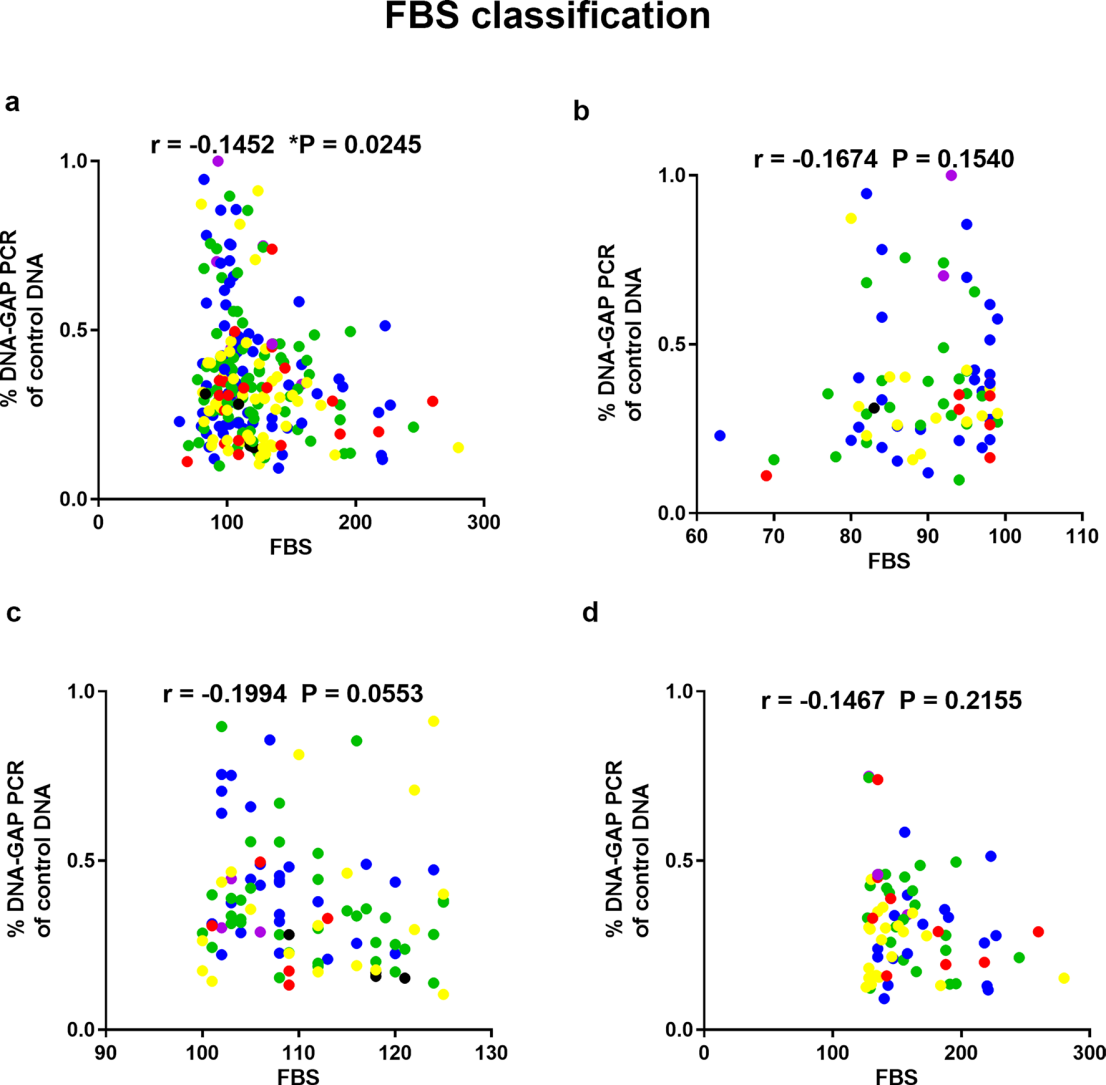
Correlation of DNA-gap number with FBS level in all samples (**a**), normal (**b**), pre-DM (**c**) and type 2 DM patients (**d**). Spearman's rank correlation (r) with P values are indicated. (*P < 0.05). The age ranges are represented by the following colors: violet (31–40 years), blue (41–50 years), green (51–60 years), yellow (61–70 years), red (71–80 years), and black (81–90 years).

Correlations between youth-DNA-gap numbers and HbA1c levels in all samples, normal, pre-DM, and type 2 DM, were reported (Fig. 2a–d) (r^2^ = − 0.2199, P = 0.0006, r^2^ = − 0.3683, P = 0.0030, r^2^ = − 0.0380, P = 0.7410, and r^2^ = − 0.3027, P = 0.0023, respectively). The results showed a correlation between youth-DNA-gap number and FBS level in all samples, normal, pre-DM, and type 2 DM (Fig. 3a–d). There was a significant negative relationship between youth-DNA-gap number and FBS level in all sample (r^2^ = − 0.1452, P = 0.0245) (Fig. 3a).

### Correlation between youth-DNA-gap number and age in all in samples, normal, pre-DM and type 2 DM

**Figure 4:**
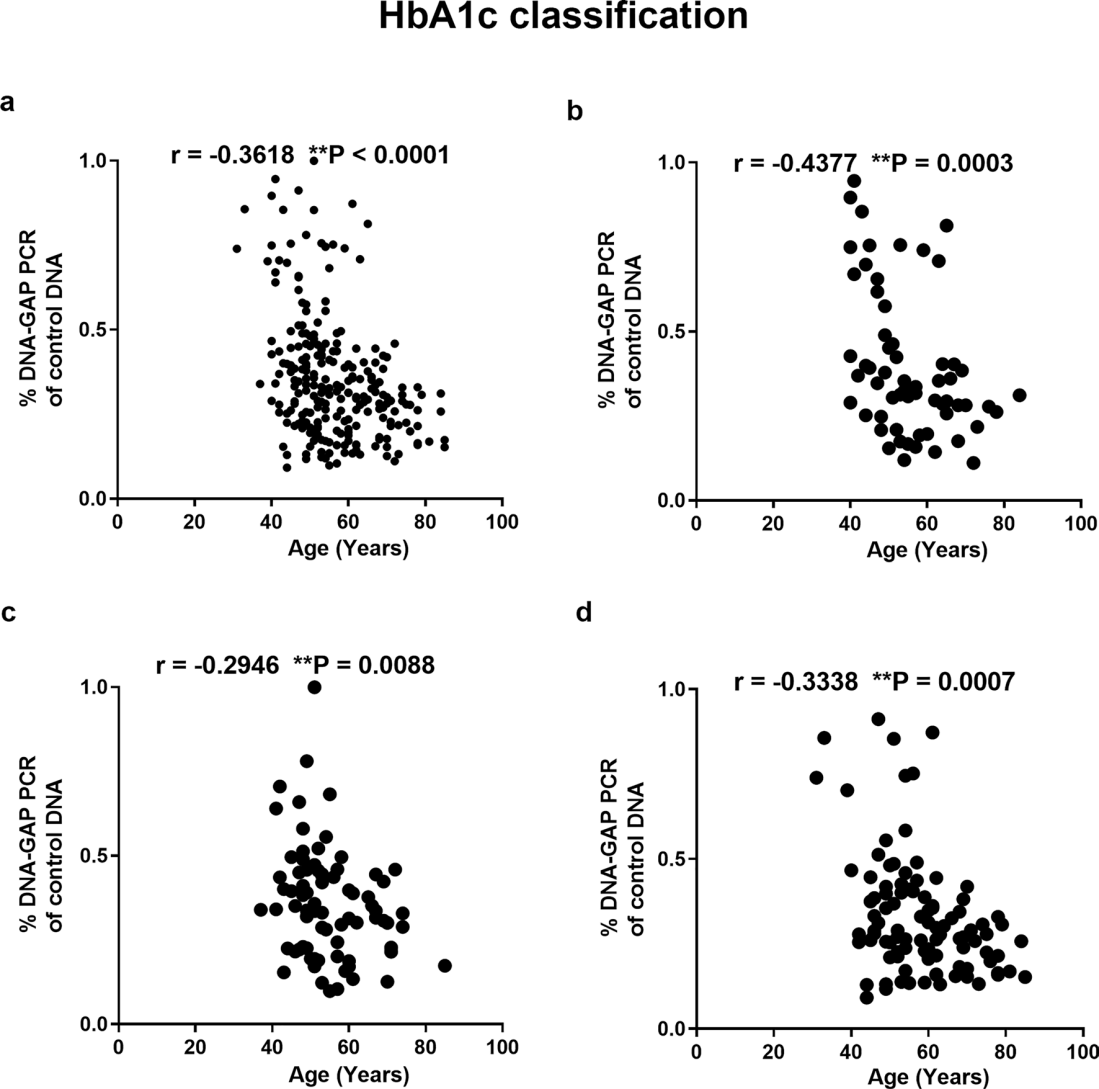
Association between the percentage of each DNA-gap and age when patients were grouped according to HbA1c levels. Correlation between % DNA-GAPs and age in all samples (**a**), normal (**b**), pre-DM (**c**), and type 2 DM (**d**). Each plot represents youth-DNA-gap levels of the patients. Spearman's rank correlation (r) with P values are indicated. **P < 0.01.

**Figure 5:**
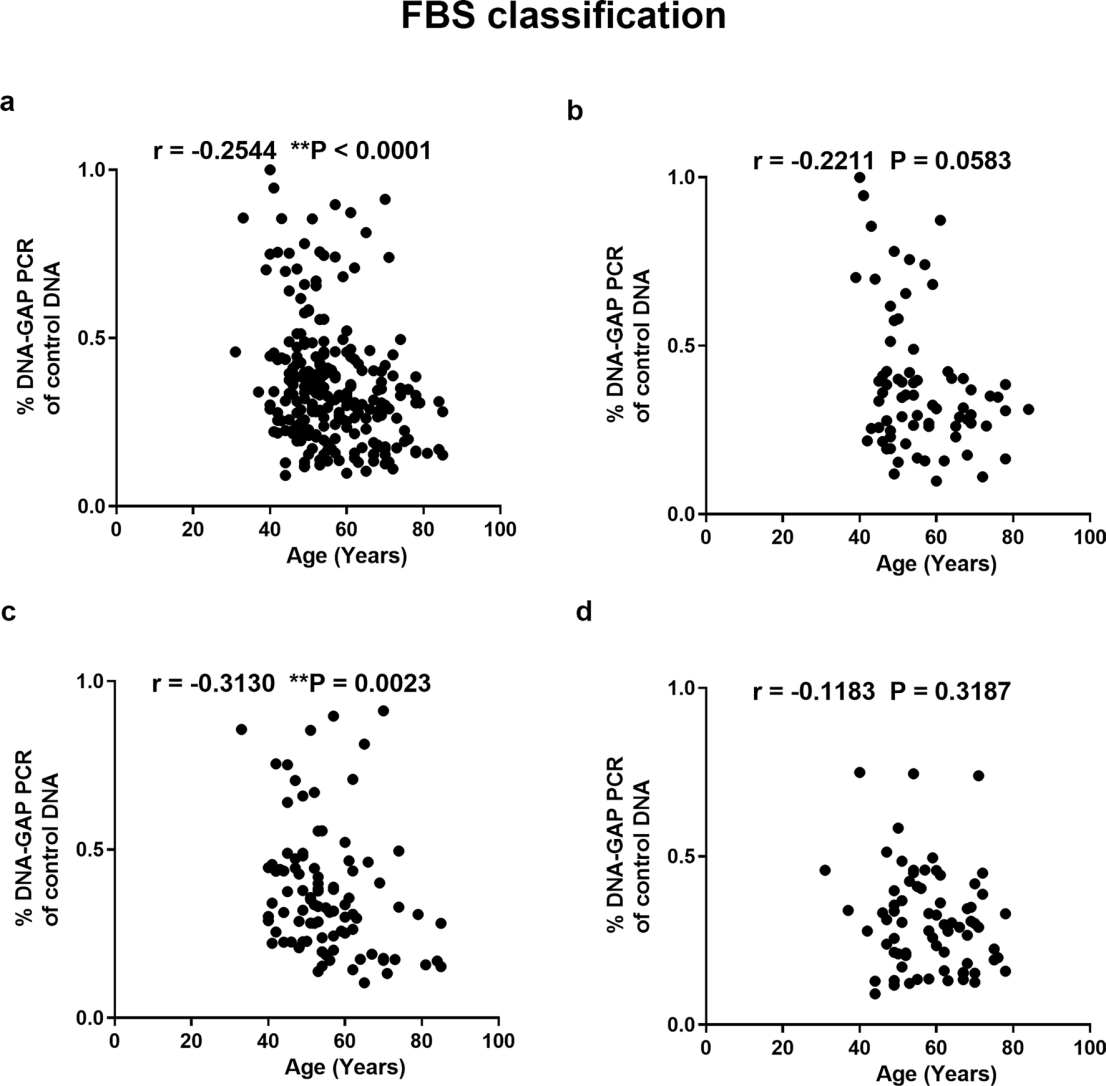
Correlation between the percentage of each DNA-gap and age in all samples (**a**), normal (**b**), pre-DM (**c**), and type 2 DM patients (**d**) when grouped according to FBS level. Spearman's rank correlation (r) with P values are indicated. **P < 0.01.

We investigated the relationship between youth-DNA-gap number and age in the the HbA1c and FBS groups. The results showed a significant negative correlation between youth-DNA-gap numbers and age in all groups (all samples, normal, pre-DM, and type 2 DM) when using HbA1c levels (Fig. 4a–d) (r^2^ = − 0.3618, P < 0.0001, r^2^ = − 0.4377, P = 0.0003, r^2^ = − 0.2946, P = 0.0088, and r^2^ = − 0.3338, P = 0.0007, respectively) and FBS levels (Fig. 5a–d) (r^2^ = − 0.2544, P < 0.0001, r^2^ = − 0.2211, P = 0.0583, r^2^ = − 0.3130, P = 0.0023, and r^2^ = − 0.1183, P = 0.3187, respectively).

**Figure 6:**
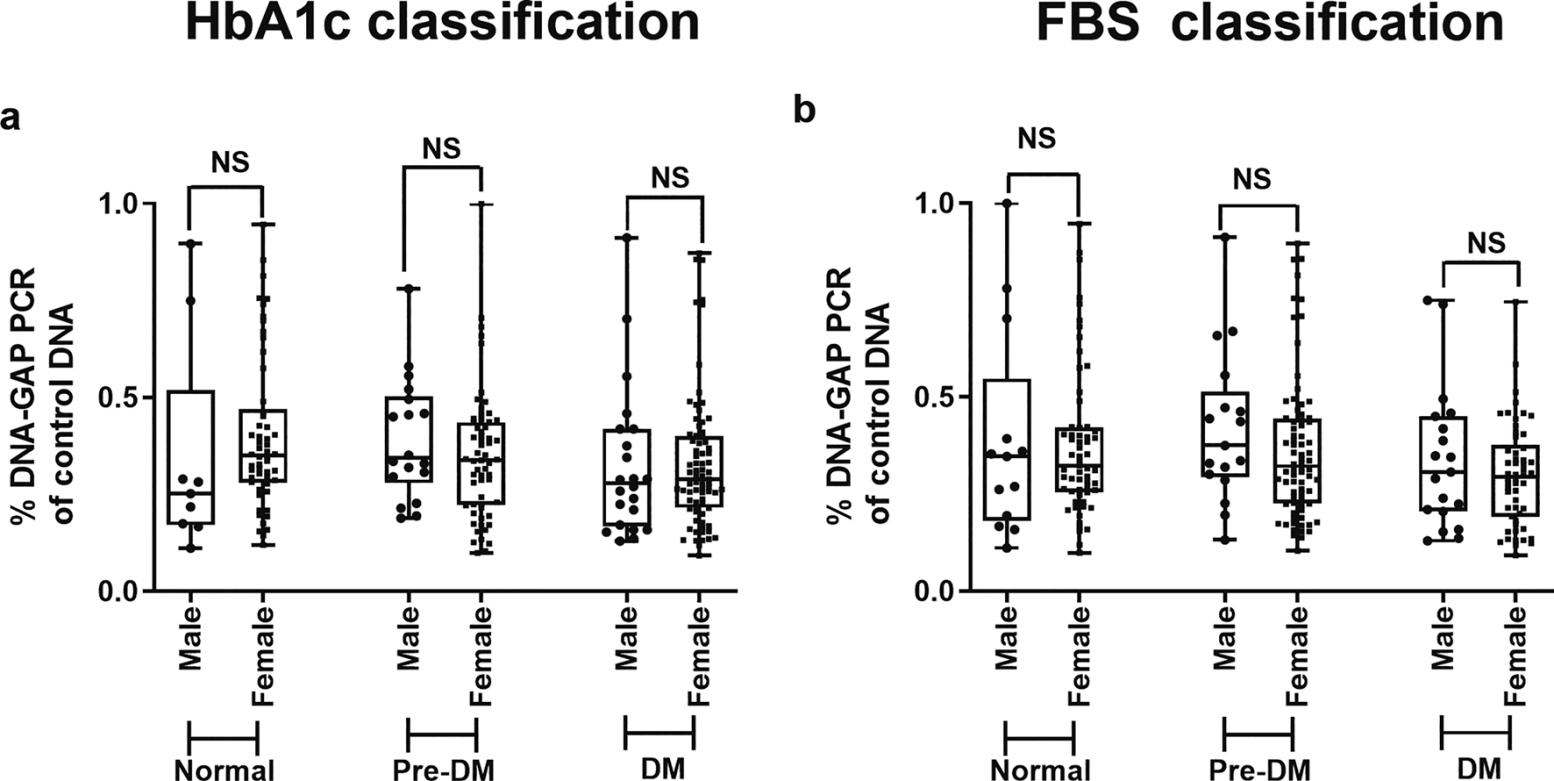
Comparisons of the percentage of DNA-gap levels between males and females in the normal, pre-DM and DM groups when grouped using the HbA1c level (**a**) and FBS level (**b**). The boxes represent the interquartile ranges (25th to 75th percentile), while the median lines represent the 50th percentile. The whiskers represent the minimum and maximum values, and all individual data points are displayed. (Mann–Whitney U test).

Here, we separated males and females into normal, pre-DM, and type 2 DM groups. The gender distribution was determined by voluntary participation, resulting in a higher number of females, which accounts for the greater representation of females in this study. Despite the difference in the number of genders, the Youth-DNA-gap numbers in males and females in the HbA1c group (Fig. 6a) and FBS group (Fig. 6b) did not differ significantly. This demonstrates that gender has no impact on the youth-DNA-gaps.

## Discussion

Previously, we established that youth-DNA gaps are epigenetic marks distributed genome-wide under the influence of AGO4, commonly binding to interspersed repetitive sequences^34,37,38^. Youth-DNA-gaps prevent DNA damage, DNA damage response, and cellular senescence^33^. By utilizing Box A of the HMGB1 plasmid to create DNA gaps, we not only reversed aging phenotypes but also successfully cured age-associated diseases in two rat aging models. Our research demonstrated that HMGB1-produced DNA gaps effectively cured conditions such as senile dementia, poor liver function, liver fibrosis, and reversed symptoms of insulin resistance, increased visceral fat, and the size of islets of Langerhans^33^. As a result, we hypothesize that the reduction of youth-DNA gaps may facilitate age-associated diseases, including type 2 DM.

In this study, we observed a reduction in the number of youth-DNA gaps in patients with type 2 DM. Naturally occurring youth-DNA gaps in the genome play a crucial role in maintaining genomic integrity and tend to decrease with aging in yeast, rats, humans, and in cells induced into senescence by chemicals^33,36–38^. Therefore, to determine whether the association between youth-DNA-gap and type 2 DM was influenced by age, we adjusted for age before comparing normal and type 2 DM patients, and the results remained significant after adjusting for age. The number of youth-DNA gaps displayed an inverse correlation with age when classified by HbA1c levels, which was more pronounced than when classified by FBS levels. Additionally, Youth-DNA-gap number inversely correlated with HbA1c levels more than FBS levels. FBS can be reduced in the short term through diet and exercise before testing, and patients only need to fast overnight for at least 8 to 16 h before the test^73^. In contrast, HbA1c is proportional to the average glucose, and erythrocytes have a lifespan of approximately 120 days^74^. As a result, HbA1c values cannot be changed in the short term, making HbA1c levels better than FBS levels for confirming type 2 DM^75^. More importantly, HbA1c levels represent polymer structure alteration due to the molecular pathogenesis of type 2 DM. The strong correlation between high HbA1c levels and low youth-DNA-gap numbers may imply a connection between reduced youth-DNA-gap numbers and molecular pathology of type 2 DM.

Interestingly, HbA1c levels are associated with other molecular aging markers. HbA1c levels are independently and inversely correlated with telomere length in peripheral blood, genome-wide methylation, and DNA methylation markers of youth^58,61,76^. Previously, our results showed the roles of the youth-DNA-gap in genomic stability and senescence prevention, and lower numbers of youth-DNA-gaps indicates progressive DNA aging^33^. Our result showed the association between a low youth-DNA-gap and high HbA1c confirmed that HbA1c represents the cellular aging biomarker of type 2 DM and supported a hypothesis that a low youth-DNA-gap may drive cellular senescence in type 2 DM. Youth-DNA-gap reduction in type 2 DM may be caused by hyperglycemia. High blood glucose (hyperglycemia) is the most important factor in the progression of type 2 DM^77–86^. Prolonged hyperglycemia significantly promotes HMGB1 release, causing an upregulation of proinflammatory cytokines and increasing the expression of oxidative production and suppression^87,88^. Glucose promotes HMGB1 release, resulting in a lack of intranuclear HMGB1. Youth-DNA-gap complexes are composed of Box A of HMGB1, which produces the DNA gap, SIRT1 deacetylated histones, and AGO4 methylated interspersed repetitive sequences^31–35^. HMGB1 produces the youth-DNA-gap, so HMGB1 release that cause decreases in intranuclear HMGB1 results in decreases in youth-DNA-gaps.

Moreover. Increased hyperglycemia is triggered in response to various cellular stresses, such as oxidative stress, inflammation, and DNA damage^89–91^. Accumulating evidence suggests that oxidative stress can directly damage DNA, resulting in the formation of DNA adducts and strand breaks^92,93^. The presence of DNA damage initiates p53 activation as a protective response^94,95^. Furthermore, p53 can directly induce tyrosine phosphorylation of insulin receptor substrate 1 (IRS1). This effect has been found to inhibit insulin signaling and impede glucose uptake. Consequently, increased DNA damage-inducing p53 activity can lead to a decrease in IRS1 expression, modifications in IRS1 phosphorylation, disruption of downstream insulin signaling pathways, and contributing to insulin resistance^96–99^. Therefore, hyperglycemia leads to an imbalance in oxidative production and suppression. This imbalance induces p53 activation, contributing to impaired insulin signaling, compromised glucose metabolism, and a decrease in intranuclear HMGB1 levels, ultimately resulting in fewer youth-DNA-gaps. Youth-DNA-gaps play a crucial role in genomic stability and senescence prevention^33^. Lower numbers of youth-DNA-gaps indicate an adverse effect on genomic stability and cell aging, which may lead to the pathogenesis of type 2 DM.

Decreased youth-DNA-gap numbers may drive DM pathogenesis. Youth-DNA-gap reduction causes DNA damage and consequences that lead to senescence, inflammation, and tissue degeneration^33^. Several studies have found that DNA damage accumulates and leads to genomic instability during the aging process and in type 2 DM patients^100–110^. A study showed that the loss of SIRT1 accelerates DNA damage in diabetes^111^. Loss of SIRT1 function also results in decreased youth-DNA-gap numbers^36^. One of the pathogenic mechanisms of type 2 DM has been proposed to be cellular dysfunction caused by senescence^112–115^. These pieces of evidence support a hypothesis that decreased youth-DNA-gap numbers promote type 2 DM pathogenesis via a DNA damage-induced senescence cascade. Therefore, we hypothesize that decreased youth-DNA-gap numbers may be associated with the molecular pathogenesis of high blood glucose, so youth-DNA-gap levels are another biomarker for monitoring the HbA1c levels in type 2 DM. Previously, we showed that Box A of the HMGB1 expression plasmid could revitalize aging cells and rats by producing Box A-produced DNA gaps. As a result, Box A increased DNA durability and stopped the DNA damage-induced senescence cascade^33^. Therefore, it is exciting and crucial to investigate whether Box A can reverse the pathogenesis of type 2 DM-associated degenerative diseases.

## Conclusions

Youth-DNA-gaps in the genome help maintain genomic stability and prevent cell aging. Our findings revealed a significant decrease in youth-DNA-gap numbers in type 2 DM patients compared to normal controls and an inverse relationship between youth-DNA-gap numbers and HbA1c levels in type 2 DM patients. Decreased youth-DNA-gap numbers may promote the deterioration of cellular function in type 2 DM patients, so youth-DNA-gaps are a promising biomarker and drug target for monitoring and treating type 2 DM tissue degeneration.

## Materials and methods

### Participants

In this study, we included 240 samples with blood glucose levels monitored using HbA1c. HbA1c values below 5.7% were considered normal, while values between 5.7 and 6.4% indicated pre-DM, and values of 6.5% or higher were indicative of type 2 DM. We divided the samples into three groups: 63 were classified as normal, 78 as pre-DM, and 99 as type 2 DM patients. Additionally, we classified the same samples based on fasting blood sugar (FBS) levels: less than 100 mg/dL was considered normal, 100 to 125 mg/dL indicated pre-DM, and 126 mg/dL or higher indicated type 2 DM. Consequently, some patients changed groups, resulting in 74 normal controls, 93 pre-DM, and 73 type 2 DM patients. All participants in these groups were monitored at the Tambon Health Promoting Hospital in Thailand, with ages ranging from 31 to 85 years. Patients with kidney or cancer conditions were excluded from the experiment. The Ethics Clearance Committee on Human Rights Related to Research Involving Human Subjects, Walailak University, Nakorn Sri Thammarat, Thailand, reviewed and approved the study. Written informed consent was provided by each participant. The study was voluntary for all the participants, and all methods were performed in accordance with the relevant guidelines and regulations.

### High-molecular-weight DNA (HMWDNA) preparation

HMWDNA was prepared to preserve the integrity of genomic DNA. Cells (approximately 1 × 10^6^ cells) were collected as previously described^33^. In brief, cells were mixed and embedded in 70 µl of 1% low-melting-point agarose (MO BIO, CA, USA), lysed and digested in a 400 µl lysis buffer (50 mM Tris pH 8.0, 20 mM EDTA, 1% sodium lauryl sarcosine, and 1 mg/ml proteinase K), and then incubated overnight at 37 °C. The next day, the digested plugs were washed with Tris–EDTA buffer 6 times for 40 min each. The cohesive end-DNA was polished using T4 DNA polymerase and dNTPs (New England Biolabs, MA, USA)^37^.

### HMWDNA preparation for DNA-GAP PCR

Plugs were incubated at 37 °C for 60 min before being washed six times with Tris–EDTA buffer for 20 min each. Ligation-mediated PCR (LM-PCR) linkers (5′-AGGTAACGAGTCAGACCACCGATCGCTCGGAAGCTTACCTCGTGGACGT-3′ and 5′-ACGTCCACGAG-3′) were prepared and ligated to polished the DNA in the plugs with T4 DNA ligase (New England Biolabs), and the plugs were incubated at room temperature for two nights. Using a QIAquick gel extraction kit, HMWDNA was extracted from the agarose plugs (Qiagen, Basel, Switzerland). HMWDNA from each plug was diluted to 20 ng/µl for DNA-GAP PCR.

### DNA-GAP measurement

The IRS-EDSB-LM-PCR or DNA-GAP PCR was performed as previously described^33^. HMWDNA was used for DNA-GAP PCR to identify EDSBs in cells using a QuanStudioTM 6 Flex Real-Time PCR system (Thermo Fisher Scientific, MA, USA). The following components were included in the PCR mix: 1× TaqmanTM Universal PCR Master mix (Applied Biosystems, CA, USA), 0.5 U HotStarTaq DNA polymerase (Qiagen, Hilden, Germany), 0.3 µM probe homologous to the 3′-linker sequence (6-fam) ACGTCCACGAGGTAAGCTTCCGAGCGA (tamra) (phosphate), 0.5 µM IRS primer (LINE-1) (5'-CTCCCAGCGTGAGCGAC-3'). EcoRV and AluI (Thermo Fisher Scientific, MA, USA) were used to digest the control DNA, which was then ligated with linkers. The following were the PCR cycle conditions: 1 cycle of 50 °C for 2 min followed by 95 °C for 10 min and 60 cycles of 95 °C for 15 s and then 60 °C for 2 min. The amount of DNA-GAP PCR in each reaction was compared to the ligated control digested DNA and reported as a percentage of EcoRV and AluI-digested genome DNA-GAP PCR amplicons (%DNA-GAP PCR of control DNA).

### Statistical analyses

The data were analyzed using SPSS statistical software. The mean ± SD and median represent the average and distributions of characteristic data in all the samples. T tests were used to determine differences in blood glucose levels between groups in matched cases using a P value threshold of 0.05. To investigate the relationship between two continuous variables, Spearman's correlation coefficient was used.

### Ethics approval and consent to participate

The study was reviewed and approved by the Ethics Clearance Committee on Human Rights Related to Researched Involving Human Subjects, Walailak University, Nakorn Sri Thammarat, Thailand. Written informed consent was obtained from each participant.

## Data Availability

All data generated or analyzed during this study are included in this published article. Further inquiries can be directed to the corresponding author.

## Abbreviations

DM: Diabetes mellitus
FBS: Fasting blood sugar
Hyperglycemia: High blood glucose levels
HbA1c: Hemoglobin A1c
Youth-DNA-gaps: Youth-associated genome-stabilizing DNA gaps
